# Dr. Orlando Webb Sutton

**Published:** 1913-06

**Authors:** 


Dr. Orlando Webb Sutton, N. Y. Electic, 1891, died at his home in Bath, April 25.

